# The MenoStim Trial: Study Protocol for a Randomised, Sham-Controlled, Double-Blinded, Pilot Clinical Trial Exploring the Neurophysiological, Cognitive, Mood and Biochemical Effects Associated with Non-Invasive Brain Stimulation During the Menopause Transition

**DOI:** 10.1136/bmjopen-2025-106745

**Published:** 2025-12-19

**Authors:** Najwa-Joelle Metri, Rocco Cavaleri, Ghufran Alhassani, Carolyn Ee, Chai K Lim, Heather M. Francis, Daniel Hochstrasser, Rose Mery Bou Merhy, Genevieve Z Steiner-Lim

**Affiliations:** 1 NICM Health Research Institute, Western Sydney University, Penrith, NSW, Australia; 2 Brain Stimulation and Rehabilitation (BrainStAR Lab), School of Health Sciences, Western Sydney University, Sydney, NSW, Australia; 3 Caring Futures Institute, College of Nursing and Health Sciences, Flinders University, Adelaide, South Australia, Australia; 4 Department of Child and Adolescent Psychiatry, Psychosomatic Medicine and Psychotherapy, Jena University Hospital, Jena, Germany; 5 German Centre for Mental Health (DZPG), Partner Site Jena, Jena, Germany; 6 School of Psychological Sciences, Macquarie University, Macquarie Park, NSW, Australia; 7 Translational Health Research Institute (THRI), Western Sydney University, Penrith, NSW, Australia; 8 Department of Psychiatry and Psychotherapy, Jena University Hospital, Jena, Germany

**Keywords:** Cognition, Transcranial Magnetic Stimulation, Depression & mood disorders, Brain, Electroencephalography, Aging

## Abstract

**Introduction:**

Intermittent theta-burst stimulation (iTBS) is a non-invasive brain stimulation technique that has been shown to improve cognition and mood when applied to certain brain structures and regions. Despite research demonstrating that iTBS may have clinical utility in treating cognitive and mood changes, no study has yet been conducted to explore the potential to modulate the neurophysiological changes that can underpin cognitive and mood changes during the menopause transition. Cognitive and psychological symptoms are commonly reported by females experiencing the menopause transition, and it is thought that these symptoms arise due to various neurophysiological, metabolic and endocrinological changes. Despite being common, there is a lack of treatments available for managing these symptoms and a scarcity of data regarding the mechanisms by which they occur.

**Methods and analysis:**

The aim of this 5-week randomised, sham-controlled, double-blinded pilot clinical trial (n=72) is to assess the underlying mechanisms of action of iTBS in females in the late menopause transition and the relationship with cognition and mood. Data will be analysed using Stata^TM^. Normality checks will guide the choice between parametric and non-parametric tests. Generalised linear models will assess within-subject and between-subject effects across timepoints, with additional regression analyses exploring associations between biomarkers, cognition and mood. Effect sizes, CIs and relevant test statistics will be reported, with significance set at p<0.05.

**Ethics and dissemination:**

The study protocol has been reviewed and ethically approved by the Western Sydney University Human Research Ethics Committee (H16200; 8 November 2024). All participants will provide written informed consent prior to enrolment. Results from this trial will be disseminated via peer-reviewed publications and conference presentations, with findings shared in accordance with open science and data transparency principles.

**ANZCTR registration number:**

ACTRN12625000030471, Australian New Zealand Clinical Trials Registry

**STRENGTHS AND LIMITATIONS OF THIS STUDY**

- Randomised, double-blinded, sham-controlled design minimises bias and enhances internal validity.
- Multimodal assessments (electroencephalogram, transcranial magnetic stimulation, cognition, mood and biomarkers) capture both mechanistic and clinical effects.
- Clearly defined late-menopause transition criteria ensure a well-characterised sample.
- A matched sham coil reproducing the sound and scalp sensations of active stimulation maintains blinding integrity.
- Single-site pilot design with a small sample limits generalisability but supports feasibility assessment.

##  Introduction

### Background and Rationale

Menopause marks the permanent cessation of menstruation and is preceded by a midlife neuroendocrine shift known as the menopause transition. While vasomotor symptoms are the most reported and studied, there is limited research on managing other prevalent symptoms. This is significant, given that 62–67% of females report subjective cognitive complaints (SCCs) during this period, such as difficulties with concentration, memory, planning and ‘brain fog’.^1^ In addition, studies show objective impairments in working memory, verbal memory and executive function compared with reproductive-age females.^2^ Survey data also indicate that 47% of menopausal females report depression and 37% experience anxiety.^3^

Cognitive and psychological symptoms throughout the menopause transition are thought to stem from neurophysiological, metabolic and endocrinological changes. A recent multimodal neuroimaging study found that menopause negatively affects white matter structure, energy metabolism and connectivity in regions responsible for higher-order cognition.^4^ Another study reported reduced glucose metabolism in key cortical areas, correlated with decreased mitochondrial cytochrome C oxidase activity in peri-menopausal and postmenopausal females.^5^ Preclinical findings further show that the shift to irregular menstrual cycling is marked by reduced bioenergetic gene expression, impaired glucose metabolism, mitochondrial dysfunction and diminished long-term potentiation.^6^ Electroencephalogram (EEG) studies have identified distinct changes in brain activity during the menopause transition. Elevated beta power during sleep in late-transition and postmenopausal females indicates hyperarousal and poor sleep quality.^7^ These data support the hypotheses that targeting brain bioenergetics and neurocognitive changes may be the key to developing novel treatments for menopause-related symptoms.

The International 2023 Practitioner’s Toolkit for Managing Menopause notes the most robust Clinical Practice Guidelines support menopausal hormone therapy (MHT) as the most effective treatment to improve vasomotor symptoms. Recent data suggest that it is still unclear whether MHT can result in improved cognitive function in midlife women due to insufficient evidence.^8^ MHT is also associated with specific adverse drug reactions and is contra-indicated in some oestrogen-dependent cancers.

The limited treatment options for managing cognitive and psychological symptoms of menopause, along with potential side effects of MHT, have driven interest in non-pharmacological alternatives that can target neurophysiological changes. Repetitive transcranial magnetic stimulation (rTMS), a non-invasive technique that uses electromagnetic fields to stimulate cortical activity, has shown promise in the treatment of psychiatric and neurological disorders. rTMS applied to certain brain regions and structures has been shown to improve cognition and mood. The dorsolateral prefrontal cortex (DLPFC) is a core region involved in executive functions including attention, working memory and emotional regulation, and so has become an area of interest in the management of cognitive and psychological symptoms. Two meta-analyses have shown that high-frequency rTMS over DLPFC can significantly improve global cognitive function.^9 10^ Systematic reviews and meta-analyses support the efficacy and acceptability of rTMS over DLPFC for treating depression.^11 12^ The non-invasive nature of rTMS and its low side effect profile suggests that the intervention may be of high clinical utility for treating the cognitive and mood symptom profiles in menopause.

Although rTMS has shown acceptable efficacy, remission and dropout rates, its standard daily treatment schedule presents feasibility challenges. Protocols typically require 10 or more 20–40-min sessions per week over several weeks, which can be time-consuming and burdensome. These practical demands, along with delays in symptom improvement, pose limitations for clinical trial recruitment and retention. Such limitations may be ameliorated through the use of theta-burst stimulation (TBS) protocols, which represent a rapid form of rTMS, which have been shown to up-regulate or down-regulate excitability in the cortex by mimicking the natural neuronal firing pattern frequencies of the brain.^13^ Preliminary research suggests that intermittent patterns of TBS (iTBS) can induce changes in cortical activity comparable to traditional rTMS in a fraction of the time (40–190 s per session). Further, recent systematic reviews have shown that iTBS can improve cognition in healthy people and people with neurological disorders^14^ and mood in people with depressive disorders.^15^

### Objectives and Trial Design

Despite research demonstrating that iTBS may have clinical utility in treating cognitive and mood changes, no such study has been conducted to explore the potential of iTBS to modulate the neurophysiological changes that can underpin cognitive and mood changes during the menopause transition. Such data are essential in identifying potential mechanisms by which to treat or prevent cognitive decline during this transition. The objective of this randomised, sham-controlled, double-blinded pilot clinical trial is to assess the underlying mechanisms of action of iTBS in females in the late menopause transition and to assess the relationship with cognition and mood.

A pilot trial design was selected due to the novelty and exploratory nature of the research question; and to assess the intervention mechanisms to determine whether the protocol will be viable in future studies that would explore treatment efficacy as the primary outcome.

## METHODS AND ANALYSIS

### Study Setting

This research will be conducted in an academic setting. Study sites include the NICM Health Research Institute, Western Sydney University (Westmead NSW 2145, Australia).

### Patient and Public Involvement (PPI)

A public involvement group was formed consisting of cognitive neuroscientists, neuropsychologists, analytical biochemists, physiotherapists and GPs experienced in cognitive assessments, TMS interventions and the management of menopause symptoms. The study methods were refined by input gained through discussions with these professionals. Females with lived experience of the menopause transition were consulted and helped with refining the study methods and participant resources. Advocacy groups, such as the Australasian Menopause Society, will provide support for recruitment and dissemination via newsletters, websites and social media. Consumers (e.g., females experiencing the menopause transition) will be consulted in the writing of lay summaries for dissemination of the trial findings to relevant patient, community and clinical groups.

### Inclusion and Exclusion Criteria

This pilot clinical trial will recruit females in the late menopausal transition stage (Stage −1 according to the STRAW+10 Criteria^16^). This cohort was chosen for two primary reasons: (a) there is evidence to suggest that the cognitive deficits reported are most pronounced during the late menopause transition and can normalise in the postmenopause period^17^; and (b) rTMS treatment responsivity in females with mood disorders is associated with a higher oestradiol/progesterone ratio.^18^ Inclusion and exclusion criteria are summarised in box 1.

Box 1 **Inclusion and Exclusion Criteria**

**Inclusion Criteria**

Females aged between 45 to 55 years who are undergoing the late menopausal transition stage according to the STRAW criteria as defined by: (a) amenorrhoea of 60 days or longer; (b) follicle-stimulating hormone (FSH) levels greater than 25 IU/L in a random blood draw and (c) self-reported vasomotor symptoms.

Females experiencing self-reported subjective cognitive complaints relative to previously normal cognitive status.

Willing to complete all study-related activities for the complete trial, including in-person assessments and remote follow-ups.

**Exclusion Criteria**

Individuals who have contraindications to TMS.

Reproductive age females; females in the late reproductive stage or the early menopausal transition and postmenopausal females.

Females who have been receiving MHT treatment for less than 3 months at the time of recruitment or plan to commence MHT treatment during the study duration.

Individuals using neuroactive medications, including anticonvulsants, antidepressants and anxiolytics.

A diagnosis of neurodegenerative, psychiatric affective, non-affective or neurological illness.

Significant cognitive impairment as deemed by scoring 17 or less on the Telephone Montreal Cognitive Assessment (T-MoCA).

Current episode of major depression as deemed by scoring 15 or greater according to the Patient Health Questionnaire-9 (PHQ-9).

High dependence on medical care due to past or current medical conditions.

Study physician discretion regarding medical status, appropriateness of participation or concern about intervention adherence.

Individuals who are not willing to follow the study protocol, eg, are not able to attend face-to-face visits or those who plan to move out of the area within the treatment period.

Individuals who are actively participating in another clinical trial(s).

Individuals who are not proficient in reading and writing in English.

### Intervention

Participants will receive five sessions of iTBS over the left DLPFC. iTBS will be delivered using a MagVenture Pro x100 including MagOption (MagVenture, Ferum, Denmark). The coil will be held tangential to the scalp with the handle pointed posterolaterally away from the midline at 45 degrees to induce a second phase current in the posterolateral to anteromedial direction.^19^ The TMS coil will be positioned through a Brainsight neuro-navigation system (Rogue Research Inc., Canada) based on anatomical landmarks in MNI space.^20^ The coil will be positioned over the left dorsolateral prefrontal cortex (DLPFC) in accordance with the BeamF3 algorithm.^21^ A fixed, multi-hinge, TMS coil armature will be used to maintain accurate coil positioning during the intervention. The coil location and orientation will be monitored throughout the procedure using the Brainsight neuro-navigation system.

Bipolar surface electrodes (Ag/AgCl) will be used to record electromyographic (EMG) activity and determine the resting motor threshold (rMT). The active electrode will be placed over the first dorsal interosseous muscle of the right hand, and the ground electrode will be placed over the right olecranon. Electromyographic signals will be amplified (32,000), band-pass-filtered (20–1000 Hz) and sampled at 2 kHz using a Power 1401 Data Acquisition System and Signal3 software (Cambridge Electronic Design, Cambridge, UK). Participants will be seated with their right arm placed on forearm pronation and elbow flexion on a pillow across their lap. rMT will be determined as the lowest stimulation intensity at which 5 out of 10 stimuli delivered to the hotspot evoked a peak-to-peak MEP of at least 0.05 mV in the resting muscle. Stimulation intensity will be delivered at 90% of the rMT, which will be assessed at the start of each iTBS session.^22^

Active treatment will consist of five sessions of iTBS over five consecutive days. On every treatment day, participants will receive five blocks of iTBS, and each block will be separated by 10 minutes.^23^ Bursts of three pulses will be delivered at 50 Hz, repeated at 200-ms intervals in trains of 2 s.^23^ 2-s trains of iTBS will be repeated every 10 seconds for a total of 600 pulses per block and 3000 pulses per session.^23^

Sham will be delivered using a dedicated sham coil that reproduces the audible clicks and superficial somatic sensations of active stimulation, without delivering magnetic pulses to the cortex. The set-up will be handled by a blinded laboratory technician, and active and sham groups will receive identical information and instructions.

### Outcomes

All outcomes were assessed at three time points and full details of the outcome measures are outlined in table 1. As this is a pilot clinical trial, primary outcomes are mechanistic and include cortical activity, assessed via electroencephalography (EEG). Paired-pulse TMS will be used to investigate activity within intracortical circuits as a co-primary outcome. Intracortical inhibition will be measured via short-interval intracortical inhibition (SICI) and long-interval intracortical inhibition (LICI).

**Table 1:** Schedule of Activities

Activity	Online Screening	Phone Screening	Baseline	Endpoint	Follow-Up
Inclusion and Exclusion Criteria	X	X			
FSH Pathology		X			
TMS Adult Safety Screen Questionnaire		X			
Telephone Montreal Cognitive Assessment		X			
EEG Measures			X	X	X
TMS Measures			X	X	X
Heart Rate Variability			X	X	X
Skin Conductance			X	X	X
Saliva Collection			X	X	X
Urine Collection			X	X	X
Memory and Cognitive Confidence Scale			X	X	X
Rey Auditory Verbal Learning Test			X	X	X
D-KEFS Verbal Fluency			X	X	X
Symbol Digit Modality Test			X	X	X
Paced Serial Addition Test			X	X	X
Conners Continuous Performance Test-3			X	X	X
Visual Analogue Scale to Evaluate Fatigue Severity			X	X	X
Patient Health Questionnaire-9		X	X	X	X
General Anxiety Disorder-7			X	X	X
Pittsburgh Sleep Quality Index			X	X	X
Menopause-Specific Quality of Life			X	X	X

D-KEFS, Delis-Kaplan Executive Function System; EEG, Electroencephalogram; FSH, Follicle Stimulating Hormone; TMS, Transcranial Magnetic Stimulation.

Secondary outcomes include changes in subjective cognitive complaints and objective changes in verbal memory, verbal learning, verbal fluency, psychomotor processing speed, working memory, and sustained attention and vigilance. The clinical trial will also assess the effects of an rTMS intervention on the psychological symptom profile as secondary outcomes, including depressive mood, anxiety, sleep quality, and quality of life.

Tertiary outcomes include changes in autonomic function, which will be assessed via skin conductance and heart rate variability (HRV). Changes in inflammatory and energetic markers and neuroactive metabolites will be measured via saliva and/or urine analysis.

### Participant Timeline

**Figure 1:**
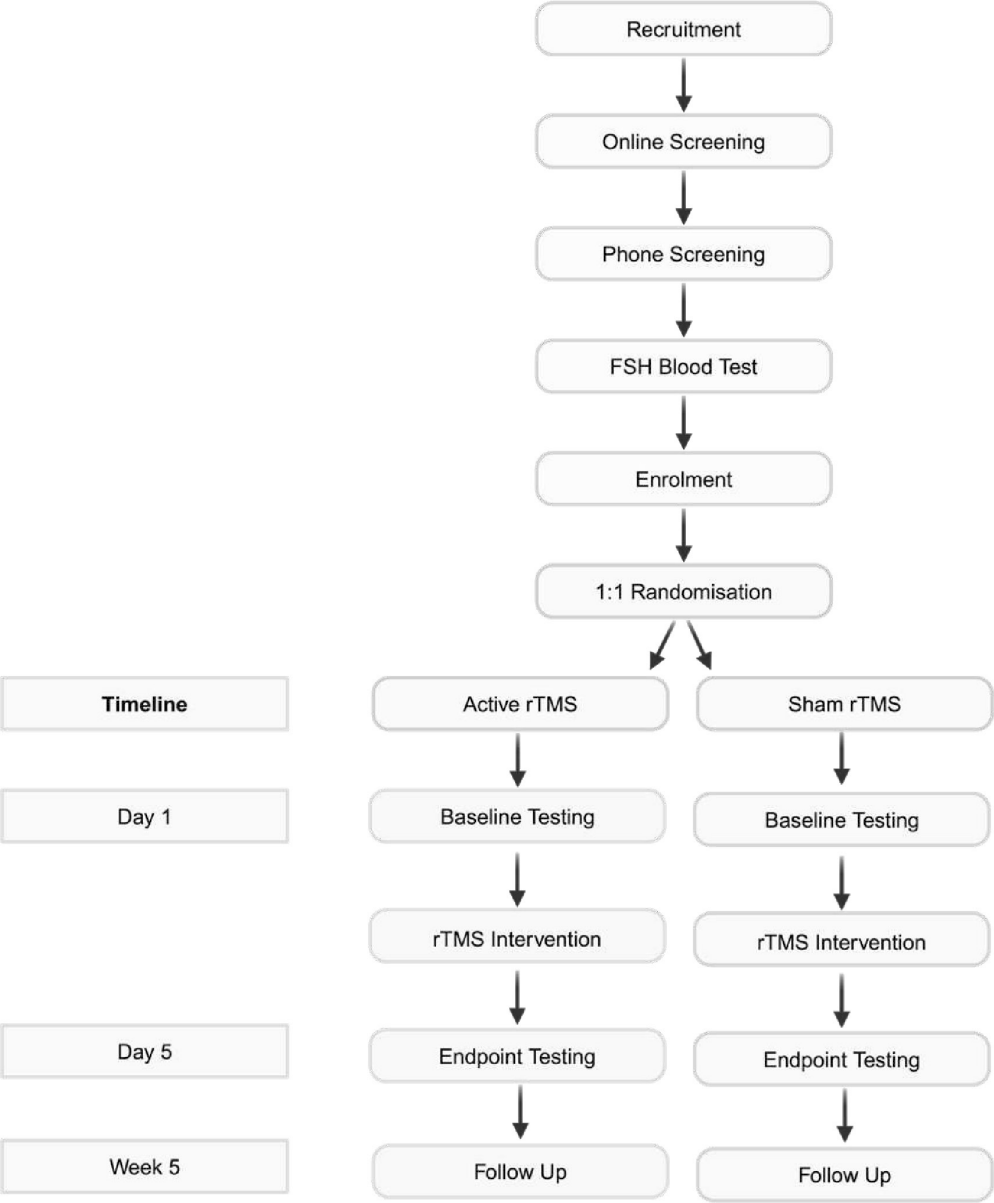
Study flow diagram. Participants will receive either active or sham rTMS treatment. Participants will be tested at three time points (baseline (Day 1), endpoint (Day 5), and follow-up (Week 5)). FSH, follicle-stimulating hormone; rTMS, repetitive transcranial magnetic stimulation

Study flow is depicted in figure 1. Participants will be tested at three time points (baseline (Day 1), endpoint (Day 5), and follow-up (Week 5)). Baseline testing will be conducted prior to the commencement of the intervention (Day 1). Participants will then be randomised into either the active or sham group. The iTBS intervention will be delivered over Days 1–5. Endpoint testing will be conducted at the conclusion of the intervention period (Day 5). Follow-up testing will be conducted 4 weeks following completion of the intervention (Week 5).

### Sample Size Determination

An exploratory sample size of 60 participants has been chosen to maximise feasibility and recruitment potential and characterise the potential mechanisms of iTBS in this pilot clinical trial. This sample size was calculated using the following calculator for binary sample sizes: https://www.johndcook.com/binary_sample_size.html. The calculator is based on the rule of thumb which assumes significance α=0.05 and type II error β=0.20 (80% power). We are assuming a potential dropout rate of 20% based on previous clinical trial experience,^24^ which requires an additional 12 participants (72 total).

### Recruitment Strategy

The primary recruitment strategy will use targeted social media advertising on the Meta platform, including Instagram and Facebook. Alternative recruitment channels include the research team’s networks, not-for-profit organisations and advocacy groups, such as Australasian Menopause Society (AMS). Interested parties will be invited to complete an online screening questionnaire to assess their initial eligibility for participation.

### Allocation

Double-blinded randomisation will be facilitated using REDCap, allowing for both concealment and randomisation. A randomisation schedule will be automatically generated by REDCap. Participants will be randomly allocated to receive rTMS or sham at a 2:1 ratio using randomly permutated blocks of 6. The trial will be double-blinded, meaning that the participants and investigator will be blinded to treatment allocation.

### Data Collection Methods

#### Electroencephalogram

Participants will be seated in an air-conditioned and sound-attenuated room with a built-in Faraday cage. Data will be acquired using the Compumedics Okti 32-channel acquisition module (Victoria, Australia), a high-definition portable EEG amplifier. Participants will be fitted with Okti EEG recording equipment and seated approximately 80 cm from a 21-inch screen with a keyboard placed in front of them. Electrodes will be positioned according to the international 10–20 montage system, covering locations including Fp1, Fpz, Fp2 and AF3, through to O2. Participants will first complete a brief electrooculogram (EOG) calibration task that will later be used for removal of EOG-related artefacts in the data.^25^ For resting-state, participants will be instructed to focus on a grey cross centred on a black background, measuring 10×10 mm, to record with their eyes open followed by eyes closed resting-state EEG for 6 min each.^26^ In both the EOG calibration and the resting-state, continuous electrophysiological data from 0 to 70 Hz will be recorded using the Compumedics CURRY nine digital signal-processing system (Victoria, Australia), referenced to Cz during active recording. Data will be digitised at 256 Hz.

Resting-state EEG data will be post-processed with standardised pipelines.^27^ Following this, aperiodic pink noise activity will be obtained due to its association with ageing and executive function and is hypothesised to reflect underlying neurobiological processes including neural spiking rates and excitatory-inhibitory balance.^28^

#### Transcranial Magnetic Stimulation (TMS)

SICI and LICI will be studied using a paired-pulse technique that employs a conditioning-test design. The test stimulus will be adjusted to evoke a motor-evoked potential (MEP) of 1 mv amplitude in the first dorsal interossei muscle of the right hand.^29^ To measure SICI, the conditioning stimulus (CS) will be adjusted at 90% of the RMT, and multiple interstimulus intervals (ISIs) will be employed. ISIs include 1, 2, 3 and 5 ms for SICI. LICI will be investigated by implementing two supra-threshold stimuli, and the CS will be adjusted at 120% of the rMT. ISIs include 50, 100 and 150 ms.^29^

#### Cognitive and Mood Assessments

All cognitive and mood assessments and their rationale are outlined in online supplemental material 1.

#### Autonomic Outcomes

Skin conductance will be recorded from the same resting-state protocol used for the EEG, using the Compumedics Okti system and UFI Bioderm Model 2701 at 0.5V.^30^ HRV will be measured using the Polar H10 sensor (Massachusetts, USA) through a 6-min eyes closed EEG task. All data will be stored on a Dell Optiplex 755 desktop.

#### Biochemical Outcomes

The biospecimen collection and processing protocol, biochemical outcomes, and analyses plans are outlined in online supplemental material 2.

### Statistical Methods

A data-driven approach will be applied to de-identified data in Stata^TM^ (StataCorp, Texas, USA). Normality checks will be performed for all variables via visual inspection of histograms to determine whether parametric or non-parametric approaches should be used. Two-tailed independent group *t*-tests with equal variances assumed will be used for all continuous variables, and χ^2^ tests will be conducted for categorical variables. Dependent variables taken at baseline and all following timepoints will be analysed for within-subject and between-subject comparisons using generalised linear models with planned simple contrasts for the within-subjects factor of time. To determine the relationship between immunological, bioenergetic and neuroimmune biomarkers and changes in cognition and mood, a logistic regression analysis will be performed.

A possible signal of efficacy will be explored via effect-size estimates of changes in tertiary outcomes (cognition and mood). Effect sizes, means, SDs and CIs will be reported. Normality checks will be performed, and data will be transformed if necessary. Where appropriate, parametric tests will be applied. A generalised linear model with fixed and random effects will be applied for the tertiary outcomes. Variables will include the between-subjects factor of group (active vs sham) and within-subjects factor of time (baseline, endpoint and follow-up). Pearson correlation coefficients (or their non-parametric counterpart) or multivariate regression involving tertiary outcomes will be conducted. All tests will be carried out one-tailed and using an alpha level of 0.05.

## DISCUSSION

This randomised, sham-controlled, double-blinded pilot trial will explore the neurophysiological, cognitive and mood effects of non-invasive brain stimulation during the late menopause transition. Building on prior research, it aims to address the evidence-practice gap in managing cognitive and mood symptoms via non-pharmacological methods.

As the first study of its kind, an exploratory sample size was selected based on previous mechanistic studies stimulating the DLPFC in healthy individuals, as well as rTMS/iTBS pilot trials reporting cognitive improvements in clinical populations (sample sizes typically 10–30). While efficacy conclusions are limited by the pilot design, the sample size allows for subgroup analyses of mechanistic outcomes. Mechanistic changes were prioritised as primary outcomes due to the exploratory nature of the study and the need to assess protocol feasibility for future efficacy trials. Results will also inform effect size estimates for adequately powered future studies.

Findings from this pilot clinical trial have the potential to advance our understanding of the underlying mechanisms that affect cognition and mood in menopause and offer the potential for new non-invasive treatments to mitigate symptoms. The successful implementation of this pilot trial could inform future trials and offer a scalable intervention to improve cognition and mood in females experiencing the late menopause transition.

## ETHICS AND DISSEMINATION

### Ethics Approval

The study protocol has been reviewed and ethically approved by the Western Sydney University Human Research Ethics Committee (H16200; 8 November 2024). Participants will be directly consented by the investigator via oral and electronic written consent. The Participant Information Sheet and Consent Form is available as online supplemental material 3. Procedures have been designed to minimise participant risk and burden, and all study activities will be conducted in accordance with the National Statement on Ethical Conduct in Human Research and relevant institutional and legal guidelines.

### Data Management and Dissemination Plan

Study data will be collected using an electronic Clinical Data Management Application (CDMA) built in REDCap, a secure, web-based platform designed for research data capture. The CMDA will be configured for real-time, anonymised data entry. Identifiable data will be encrypted or password-protected. Each participant will be assigned a unique REDCap study ID, to which all data will be securely linked. Downloaded data will be stored on WSU’s secure network in password-protected files. Data will be retained for 15 years following study completion or publication. Investigators must permit study-related monitoring, audits, ethics review and regulatory inspections and provide access to source documents. No records may be destroyed during the retention period without the written approval of the sponsor. The anonymised final dataset will be kept indefinitely to facilitate data sharing such as mega-analysis.

This trial follows the guidance published by the NHMRC: Safety Monitoring and Reporting in Clinical Trials Involving Therapeutic Goods. The investigator and medical monitor will monitor all participants for AEs during the study, and all AEs reported will be recorded in the Case Report Form from the first day of intervention through the follow-up period (see figure 1). AEs will be reported to the WSU HREC via the Annual Report. Serious adverse events (SAEs) will be reported to the WSU HREC within 24 hours of awareness. Following an initial report, the investigator will continue monitoring the participant at subsequent visits or contacts. SAEs will be followed by the medical monitor until resolution, stabilisation, explanation or loss to follow-up.

Findings from this study will be disseminated via publication in peer-reviewed open-access journals, presentation at national and international conferences, and communication with relevant stakeholder groups. Where appropriate, summary results may be shared with participants in plain-language format. The anonymised dataset and relevant study materials may also be made available on reasonable request and in accordance with FAIR data principles (Findable, Accessible, Interoperable and Reusable).

## Supplementary material

10.1136/bmjopen-2025-106745 online supplemental file 1

10.1136/bmjopen-2025-106745 online supplemental file 2

10.1136/bmjopen-2025-106745 online supplemental file 3

## Data Availability

No data are available.

## References

[1] Reuben R, Karkaby L, McNamee C (2021). Menopause and cognitive complaints: are ovarian hormones linked with subjective cognitive decline?. Climacteric.

[2] Kantarci K, Tosakulwong N, Lesnick TG (2018). Brain structure and cognition 3 years after the end of an early menopausal hormone therapy trial. Neurology (ECronicon).

[3] Soares CN (2013). Depression in Peri- and Postmenopausal Women: Prevalence, Pathophysiology and Pharmacological Management. *Drugs Aging*.

[4] Mosconi L, Berti V, Dyke J (2021). Menopause impacts human brain structure, connectivity, energy metabolism, and amyloid-beta deposition. Sci Rep.

[5] Mosconi L, Berti V, Quinn C (2017). Perimenopause and emergence of an Alzheimer’s bioenergetic phenotype in brain and periphery. PLoS One.

[6] Yin F, Yao J, Sancheti H (2015). The perimenopausal aging transition in the female rat brain: decline in bioenergetic systems and synaptic plasticity. Neurobiol Aging.

[7] Campbell IG, Bromberger JT, Buysse DJ (2011). Evaluation of the association of menopausal status with delta and beta EEG activity during sleep. Sleep.

[8] Andy C, Nerattini M, Jett S (2024). Systematic review and meta-analysis of the effects of menopause hormone therapy on cognition. Front Endocrinol (Lausanne).

[9] Chou Y-H, Ton That V, Sundman M (2020). A systematic review and meta-analysis of rTMS effects on cognitive enhancement in mild cognitive impairment and Alzheimer’s disease. Neurobiol Aging.

[10] Zhang T, Sui Y, Lu Q (2022). Effects of rTMS treatment on global cognitive function in Alzheimer’s disease: A systematic review and meta-analysis. Front Aging Neurosci.

[11] Berlim MT, van den Eynde F, Tovar-Perdomo S (2014). Response, remission and drop-out rates following high-frequency repetitive transcranial magnetic stimulation (rTMS) for treating major depression: a systematic review and meta-analysis of randomized, double-blind and sham-controlled trials. Psychol Med.

[12] De Risio L, Borgi M, Pettorruso M (2020). Recovering from depression with repetitive transcranial magnetic stimulation (rTMS): a systematic review and meta-analysis of preclinical studies. Transl Psychiatry.

[13] Diamond DM, Dunwiddie TV, Rose G (1988). Characteristics of hippocampal primed burst potentiation in vitro and in the awake rat. J Neurosci.

[14] Zheng B (2024). The effect of intermittent theta burst stimulation for cognitive dysfunction: a meta-analysis. Brain Inj.

[15] Kishi T, Ikuta T, Sakuma K (2024). Theta burst stimulation for depression: a systematic review and network and pairwise meta-analysis. Mol Psychiatry.

[16] Harlow SD, Gass M, Hall JE (2012). Executive Summary of the Stages of Reproductive Aging Workshop + 10: Addressing the Unfinished Agenda of Staging Reproductive Aging. *The Journal of Clinical Endocrinology & Metabolism*.

[17] Greendale GA, Huang M-H, Wight RG (2009). Effects of the menopause transition and hormone use on cognitive performance in midlife women. Neurology (ECronicon).

[18] Huang C-C, Wei I-H, Chou Y-H (2008). Effect of age, gender, menopausal status, and ovarian hormonal level on rTMS in treatment-resistant depression. Psychoneuroendocrinology.

[19] Gaudeau-Bosma C, Moulier V, Allard A-C (2013). Effect of two weeks of rTMS on brain activity in healthy subjects during an n-back task: a randomized double blind study. Brain Stimul.

[20] Nauczyciel C, Hellier P, Morandi X (2011). Assessment of standard coil positioning in transcranial magnetic stimulation in depression. Psychiatry Res.

[21] Mann SK, Malhi NK (2021). Repetitive transcranial magnetic stimulation.

[22] Rossini PM, Barker AT, Berardelli A (1994). Non-invasive electrical and magnetic stimulation of the brain, spinal cord and roots: basic principles and procedures for routine clinical application. Report of an IFCN committee. Electroencephalogr Clin Neurophysiol.

[23] Moukhaiber N, Summers SJ, Opar D (2023). The Effect of Theta Burst Stimulation Over the Primary Motor Cortex on Experimental Hamstring Pain: A Randomized, Controlled Study. J Pain.

[24] Steiner‐Lim GZ (2023). A randomized, double‐blind, placebo‐controlled, parallel‐group 12‐week pilot phase II trial of SaiLuoTong (SLT) for cognitive function in older adults with mild cognitive impairment. Alzheimer’s & Dementia: Translational Research & Clinical Interventions.

[25] Croft RJ, Barry RJ (2000). EOG correction of blinks with saccade coefficients: a test and revision of the aligned-artefact average solution. Clin Neurophysiol.

[26] Steiner GZ, Fernandez FM, Coles M (2019). Interrogating the Relationship Between Schizotypy, the Catechol-O-Methyltransferase (COMT) Val158Met Polymorphism, and Neuronal Oscillatory Activity. *Cereb Cortex*.

[27] Cave AE, De Blasio FM, Chang DH (2025). Eyes-open and eyes-closed EEG of older adults with subjective cognitive impairment versus healthy controls: A frequency principal components analysis study. Brain Res.

[28] Donoghue T, Haller M, Peterson EJ (2020). Parameterizing neural power spectra into periodic and aperiodic components. Nat Neurosci.

[29] Benussi A, Dell’Era V, Cantoni V (2020). TMS for staging and predicting functional decline in frontotemporal dementia. Brain Stimul.

[30] Steiner GZ, Barry RJ (2014). The mechanism of dishabituation. Front Integr Neurosci.

