# Supplementary material for: The MenoStim Trial: Study Protocol for a Randomised, Sham-Controlled, Double-Blinded, Pilot Clinical Trial Exploring the Neurophysiological, Cognitive, Mood and Biochemical Effects Associated with Non-Invasive Brain Stimulation During the Menopause Transition
Source: BMJ Open. 2025 Dec 19;15(12):e106745. doi: 10.1136/bmjopen-2025-106745 (PMC12716591; doi:10.1136/bmjopen-2025-106745)
Supplement: online supplemental file 1 [file bmjopen-15-12-s001.docx]

Cognitive Assessments

The Memory and Cognitive Confidence Scale is a self-report measure that measures a range of beliefs about memory and cognitive abilities, consisting of four individual subscales: (a) General Memory; (b) Decision Making; (c) Concentration and Attention; and (d) High Standards. This scale has been found to have sufficient internal consistency for each subscale and the overall score and it has been previously used in menopause populations [1]. Verbal learning and memory will be assessed via the Rey Auditory Verbal Learning Test [2], which has been previously used in a study which assessed the relationship between SCCs and objective cognitive deficits in females experiencing the menopause transition, as well as contentment with their cognitive abilities [3]. Verbal fluency will be assessed via the D-KEFS Verbal Fluency (Letter Fluency and Category Fluency) [4], which has been validated in individuals aged 8 to 89 years and is accepted as a valid measure of fluent productivity in the verbal domain. Psychomotor processing speed will be assessed via the Symbol Digit Modality Test [5], which has been validated in cohorts of similar age to those being recruited in this clinical trial, has acceptable test-retest reliability, and alternate forms [6]. Working memory will be assessed via the Paced Serial Addition Test, which has high levels of internal consistency and test-retest reliability [7]. Sustained attention and vigilance will be assessed via the Conners Continuous Performance Test-3, a task-oriented computerised assessment with diagnostic confidence [8]. State fatigue before and after completing cognitive assessments will be assessed via the Visual Analogue Scale to Evaluate Fatigue Severity, which places fewer restrictions on the range of responses available to participants, in contrast to other discrete scales [9].

Mood Assessments

Depressive symptoms will be assessed via the Patient Health Questionnaire-9 [10], a brief depressive symptom scale. Anxiety symptoms will be assessed via the General Anxiety Disorder-7 [11], a brief instrument used in primary care settings that has been validated in numerous menopause cohorts [12-14]. Sleep quality and disturbances will be assessed via the Pittsburgh Sleep Quality Index, a self-report questionnaire that assesses sleep quality over a one-month interval that has shown strong reliability and validity in non-clinical samples [15]. The Menopause-Specific Quality of Life (MENQOL) questionnaire will be used to measure changes in quality of life [16]. The MENQOL has four domains (vasomotor, physical, psychosocial, and sexual) and has been used extensively in clinical and epidemiological research over two decades [17].

References

1. Nedeljkovic, M. and M. Kyrios, *Confidence in memory and other cognitive processes in obsessive–compulsive disorder.* Behaviour Research and Therapy, 2007. **45**(12): p. 2899-2914.

2. Schmidt, M., *Rey auditory verbal learning test*. 1996: Western Psychological Services Los Angeles.

3. Unkenstein, A.E., et al., *Understanding women's experience of memory over the menopausal transition: subjective and objective memory in pre-, peri-, and postmenopausal women.* Menopause, 2016. **23**(12): p. 1319-1329.

4. Delis, D.C., E. Kaplan, and J.H. Kramer, *Delis-Kaplan executive function system.* Assessment, 2001.

5. Smith, A., *Symbol digit modalities test.* The Clinical Neuropsychologist, 1973.

6. Benedict, R.H., et al., *Validity of the Symbol Digit Modalities Test as a cognition performance outcome measure for multiple sclerosis.* Multiple Sclerosis Journal, 2017. **23**(5): p. 721-733.

7. Fos, L.A., et al., *Paced Visual Serial Addition Test: an alternative measure of information processing speed.* Applied Neuropsychology, 2000. **7**(3): p. 140-146.

8. Conners, C.K., *Conners' continuous performance test*. 2000: Multi-Health Systems North Tonawanda NY.

9. Shahid, A., et al., *Visual analogue scale to evaluate fatigue severity (VAS-F)*, in *STOP, THAT and one hundred other sleep scales*. 2011, Springer. p. 399-402.

10. Levis, B., A. Benedetti, and B.D. Thombs, *Accuracy of Patient Health Questionnaire-9 (PHQ-9) for screening to detect major depression: individual participant data meta-analysis.* bmj, 2019. **365**.

11. Spitzer, R.L., et al., *A brief measure for assessing generalized anxiety disorder: the GAD-7.* Archives of internal medicine, 2006. **166**(10): p. 1092-1097.

12. Ai, F., et al., *Screening for general anxiety disorders in postmenopausal women with symptomatic pelvic organ prolapse.* Climacteric, 2018. **21**(1): p. 35-39.

13. Sahingoz, M., F. Uguz, and K. Gezginc, *Prevalence and related factors of mood and anxiety disorders in a clinical sample of postmenopausal women.* Perspectives in psychiatric care, 2011. **47**(4): p. 213-219.

14. Bromberger, J.T., et al., *Does risk for anxiety increase during the menopausal transition? Study of women’s health across the nation.* Menopause, 2013. **20**(5): p. 488-495.

15. Mollayeva, T., et al., *The Pittsburgh sleep quality index as a screening tool for sleep dysfunction in clinical and non-clinical samples: A systematic review and meta-analysis.* Sleep Medicine Reviews, 2016. **25**: p. 52-73.

16. Hilditch, J.R., et al., *A menopause-specific quality of life questionnaire: development and psychometric properties.* Maturitas, 1996. **24**(3): p. 161-75.

17. Sydora, B.C., et al., *Use of the Menopause-Specific Quality of Life (MENQOL) questionnaire in research and clinical practice: a comprehensive scoping review.* Menopause, 2016. **23**(9): p. 1038-1051.
