# Supplementary material for: The MenoStim Trial: Study Protocol for a Randomised, Sham-Controlled, Double-Blinded, Pilot Clinical Trial Exploring the Neurophysiological, Cognitive, Mood and Biochemical Effects Associated with Non-Invasive Brain Stimulation During the Menopause Transition
Source: BMJ Open. 2025 Dec 19;15(12):e106745. doi: 10.1136/bmjopen-2025-106745 (PMC12716591; doi:10.1136/bmjopen-2025-106745)
Supplement: online supplemental file 2 [file bmjopen-15-12-s002.docx]

Biochemical Outcomes

500 μL of saliva will be collected into 15mL Eppendorf tubes through passive drool at three timepoints during baseline and endpoint testing, and once during follow-up testing. Participants will be advised that they should spit into the jar until the amount of liquid saliva (not bubbles) reached the 0.5 mL fill line. Mid-stream urine will be collected into a sterile 50 mL Sarstedt collection container twice during baseline and endpoint testing, and once during follow-up testing. Samples will then be centrifuged for 10 minutes at 2500 rpm and 4 °C. Following centrifuging, 500 μL of the contents of the supernatant layer was aliquoted into 1500 μL Eppendorf tubes using 1000 μL pipette tips.

All enzyme-linked immunosorbent assays (ELISA) will be purchased from Abcam (Cambridge, UK). Inflammatory cytokines CRP, IL-2, and TNF-alpha will be measured in serum samples, as rTMS has been known to affect dynamic patterns of these markers in people living with depression and are known to be potential effect modifiers [1]. CRP will be measured in the serum using the Human CRP ELISA Kit. IL-2 will be profiled using the Human IL-2 ELISA Kit. TNF-alpha will be measured via the Human TNF Alpha ELISA Kit. We will also be measuring energetic markers in the serum, chosen based on the bioenergetic hypotheses of menopause [2]. ATP will be profiled using the ATP Assay Kit (Colorimetric/Fluorometric). NAD and NADH will be profiled using the NAD/NADH Assay Kit. We will be profiling neurometabolic and neuroimmune markers related to the kynurenine pathway (KP). All reagents used will be of analytical mass spectrometry-grade and purchased from ChemSupply (NSW, Australia).

References

1. Wang, Q., et al., *Inflammatory cytokines changed in patients with depression before and after repetitive transcranial magnetic stimulation treatment.* Frontiers in Psychiatry, 2022. **13**: p. 925007.

2. Mosconi, L., et al., *Menopause impacts human brain structure, connectivity, energy metabolism, and amyloid-beta deposition.* Scientific Reports, 2021. **11**(1): p. 10867.
