# Supplementary material for: The MenoStim Trial: Study Protocol for a Randomised, Sham-Controlled, Double-Blinded, Pilot Clinical Trial Exploring the Neurophysiological, Cognitive, Mood and Biochemical Effects Associated with Non-Invasive Brain Stimulation During the Menopause Transition
Source: BMJ Open. 2025 Dec 19;15(12):e106745. doi: 10.1136/bmjopen-2025-106745 (PMC12716591; doi:10.1136/bmjopen-2025-106745)
Supplement: online supplemental file 3 [file bmjopen-15-12-s003.pdf]

WESTERN SYDNEY
UNIVERSITY

Participant Information Sheet – Medical (Extended)

Project Title:

Exploring the Neurophysiological, Cognitive, Mood, and Biochemical Effects Associated with Non-Invasive Brain Stimulation During the Menopause Transition: A Randomised, Sham-Controlled, Double-Blinded, Pilot Controlled Clinical Trial (The MenoStim Trial)

Project Summary:

You are invited to participate in a research study being conducted by Ms Joelle Metri (PhD Candidate, NICM Health Research Institute) under the supervision of Associate Professor Genevieve Steiner-Lim (NHMRC Emerging Leadership Fellow, NICM Health Research Institute).

The aim of this research is to investigate how a potential treatment works for the cognitive and mood symptoms experienced during menopause through a form of non-invasive brain stimulation. You have likely heard of the common menopause symptoms, like hot flushes, night sweats, and sleep disturbances. These symptoms are called vasomotor symptoms, and most treatment options for menopause symptoms focus on symptoms like these. However, menopause can affect our memory, thinking, and mood. These symptoms are also common, however, there is a lack of treatment options when compared to the vasomotor symptoms. This is why we are doing research into potential treatment options for the memory, thinking, and mood symptoms that are common in menopause.

Transcranial magnetic stimulation (TMS) is a technique that can alter activation in certain areas of the brain by using magnetic stimulation. This stimulation is non-invasive, which means it is applied to the top of your scalp. The stimulation then travels through the scalp and into the brain. Specific forms of TMS, like intermittent theta-burst stimulation (iTBS) are promising treatments for depression, memory, and thinking. Even though it is promising, no one has investigated whether iTBS can improve these symptoms in people going through menopause. This is what we refer to as an evidence-practice gap.

This research will address this evidence-practice gap by using non-invasive brain stimulation in the form of iTBS to test how it works and whether it can improve cognition and mood in females going through the late menopause transition. This research will be conducted in the form of a 5-week randomised, sham-controlled, double-blinded pilot clinical trial.

How is the study being paid for?

This study is funded by the Australian Government Research Training Program, a stipend provided to support Australian students undertaking postgraduate research degrees. The study is also funded by an NHMRC Investigator Grant awarded to Associate Professor Genevieve Steiner-Lim (APP1195709).

What will I be asked to do?

Initially, you will go through a screening process to ensure that you're a good fit for the clinical trial. The first step is an online form that will ask some questions regarding your eligibility. This screening process is important, because this type of treatment is not suitable for people with certain conditions. If we think you may be eligible for the clinical trial based on your response to the online questions, we will invite you for a phone screening interview. If we still believe that you will be a good fit for the clinical trial, you will be asked to take a commercial pathology test via DHM Pathology. The reason that we ask this of you is to check levels of Follicle Stimulation Hormone, which is a hormone that can tell us about your reproductive health.

If you are found to be eligible, you will be enrolled and invited to the School of Health Sciences at Western Sydney University (Campbelltown Campus) or NICM Health Research Institute at Western Sydney University (Westmead Campus). You will be consented to the most recent version of the Participant Information Sheet and Consent Form (PISCF).

Once you've been enrolled into the clinical trial, you will be randomly allocated into either the active treatment or sham group. This clinical trial is double-blinded, which means that you and the investigators won't know whether you're receiving the active or sham treatment.

You will be required to visit either the School of Health Sciences (Campbelltown) or the NICM Health Research Institute (Westmead) every day consecutively for 5 days to receive treatment (Week 1). Sessions will be available from Monday to Saturday, and all your sessions must be delivered in one location only (either Campbelltown or Westmead). After your fifth and final session, you will then have to visit us again at the precise 4-week mark (Week 5). You will be asked to undertake tests related to your memory, thinking, and mood at three timepoints (baseline, endpoint, and follow up). Baseline testing will be conducted prior to the commencement of the first treatment session. Endpoint testing will be conducted at the conclusion of Week 1. Follow-up testing will be conducted at Week 5. Study duration (including follow up) will be 5 weeks in total. During each testing session, we will also be taking a recording of your brain activity using electroencephalogram (EEG), transcranial magnetic stimulation (TMS), heart rate variability (HRV), skin conductance, and pressure-pain threshold.

You will also be asked to provide urine and saliva samples at the three testing timepoints (baseline, endpoint, and follow up). Specifically, you will be asked to provide two urine and three saliva samples at baseline and endpoint, and one urine/saliva sample at follow up. We will use your samples to assess changes in inflammatory, energetic, and neuroimmune markers. Since you are asked to agree to extended consent, we will be storing these serum samples indefinitely. The reason for this is because we might use the samples in future, related studies. For example, we may later analyse the samples to check for other important markers in the menopause transition, like certain metabolites. If we decide to do this in the future, the new study will be subject to further ethical review by the Western Sydney University Human

Research Ethics Committee, and your privacy and confidentiality will be maintained.

How much of my time will I need to give?

Pre-screening will be conducted remotely and is expected to take up to half-an-hour including an online survey and a phone call. You will then be asked to visit a commercial DHM pathology practice to receive a blood test, which will require approximately half-an-hour of your time.

If you are enrolled in the trial, you will be asked to undergo testing at three timepoints (Day 1, Day 5, and Follow Up). Each of the testing sessions is expected to take approximately 2–3 hours. Additionally, you will be expected to visit either the School of Health Sciences (Campbelltown) or the NICM Health Research Institute (Westmead) for 5 consecutive sessions of iTBS treatment over 1 week. Each session will take approximately 1 hour (1 hour per day on Days 2, 3, and 4).

The total interactions with the research team over the 5-week period (including screening) will be around 10–13 hours.

Additionally, we will ask you to record any signs and symptoms you may feel in an electronic diary throughout the course of the trial. The electronic diary will be accessible via a link to REDCap, a secure web application that we use to build and manage online surveys and databases for our clinical trials. In REDCap, you can report any adverse events you might be experiencing, and this report will be immediately sent to our study physician for evaluation.

What benefits will I, and/or the broader community, receive for participating?

To minimise travel expenses for face-to-face appointments, you will be reimbursed $150.00 in total and provided with free parking and refreshments at these appointments. If our thoughts about this potential treatment are proven correct, participants in the active treatment group may experience improvements in memory, thinking, and mood from the study intervention. Should the study have a positive result, then this research will provide evidence on the efficacy of a new intervention to treat the cognitive and mood symptoms associated with menopause, for which there are currently no effective therapies.

However, it is important to note that this is a pilot study, meaning it is one of the first studies of its kind and any therapeutic effect cannot be guaranteed. Also, you may be allocated to the placebo group, and you or the research team will not know which group you have been assigned to. The reason we adopted this approach is to minimise bias within the clinical trial design. Participants will not have access to the active intervention beyond the period of the study, even those that were allocated to the sham group. Therapeutic benefits, and potentially access to the intervention, may become known in the future following additional research studies that follow-on from the findings of this study.

Will the study involve any risk or discomfort for me? If so, what will be done to rectify it?

It is not anticipated that participation in this project will cause harm to participants. There are few risks associated with the study and the appropriate steps have been taken to minimise the risk of any discomfort to the participant. Risks are summarised below.

a. Potential side effects of TMS include seizure induction; transient acute hypomania induction; syncope; transient headache, local pain, neck pain, toothache, paraesthesia; transient hearing changes; and burns from scalp electrodes. Side effects are rare and TMS is considered a safe and tolerable intervention when participants are appropriately screened. To minimise the risk of adverse events, individuals who have contraindications to TMS will be identified during the screening phase and will be excluded from the study. In the unlikely event of a first-time seizure in a participant, standard emergency procedures will be implemented. All research investigators and assistants hold a current first aid certificate and are well-versed in standard emergency procedures. Appropriate first aid will be provided, and an ambulance called.

b. The electrical activity in the brain will be measured through EEG. EEG is non-invasive, with minimal discomfort involved. The fitting of the electrode cap to participants' heads may be associated with a small amount of discomfort. Discomfort will be minimised by ensuring that the cap is the correct size, and the researcher will continually check in with the participants throughout the cap fitting process to ensure that the cap remains comfortable.

c. Participants may feel apprehensive about the intended use of their samples. Participants will be assured that analysis will be targeted towards a group of related metabolites that are known to be related to inflammation and energetics. The investigator will store the samples in a secure storage space with adequate measures to protect confidentiality.

d. Some participants may share experiences or information which may be negative or distressing (for example, mental or cognitive status, medical history). We will advise you to let us know if any of the questions asked trigger a response which causes distress. Distressed participants will be advised to contact HealthDirect on 1800 022 222, as suggested by the Australian Menopause Society. We may also encourage you to seek assistance that day from your general practitioner.

e. Appropriate social distancing will be maintained where possible, and PPE will be provided and worn to reduce the likelihood of the transmission of COVID-19, as required.

Considering the measures taken to minimise risk to participants in the study, the potential risks identified in associated with the potential treatment are justified by the anticipated benefits that may be afforded to participants experiencing cognitive and mood changes in the menopause transition.

If you do experience any negative signs and symptoms throughout the course of the study, we will ask you to record them in your REDCap diary for evaluation by our study physician.

How do you intend to publish or disseminate the results?

It is anticipated that the results of this research project will be published and/or presented in a variety of forums. In any publication and/or presentation, information will be provided in such a way that the participant cannot be identified, except with your permission.

The data collected from you and other participants will be deidentified and stored with a participant code so that there will be no identifiable information used in the dataset, apart from age and sex, to ensure that your confidentiality is maintained. Data from your baseline testing session may be used in future studies.

The findings of the research will be published in academic journals and/or discussed/ displayed in conference presentations. It will also be disseminated to the wider community through non-academic channels such as via social media. In any case, only group information and trends will be presented. No reference will ever be made to individual results, or individual participants.

Will the data and information that I have provided be disposed of?

Please be assured that only the researchers will have access to the raw data you provide. However, your data may be used in other related projects for an extended period. Future related projects may include follow-up projects to the current pilot clinical trial, that may have a larger sample size or different treatment intervention, for example, a longer intervention. Please note that the minimum retention period of data collected during clinical trials is 15 years for adults following completion of the trial. We intend to keep a copy of the deidentified dataset indefinitely for future research purposes.

Can I withdraw from the study?

Participation is entirely voluntary, and you are not obliged to be involved. If you do participate you can withdraw at any time without giving reason by contacting the investigator. Whatever your decision, it will not affect your medical treatment or your relationship with the medical and research staff. If you do choose to withdraw any information that you have provided will be deleted.

Can I tell other people about the study?

Yes, you can tell other people about the study by providing them with the investigatory team's contact details. They can contact the study staff to discuss their participation in the research project and obtain an information sheet.

What if I require further information?

Please contact a member of our investigator team should you wish to discuss the research further before deciding whether to participate:

For all general enquiries, please contact:

Ms Joelle Metri; phone 0411 622 021; email: j.metri@westernsydney.edu.au

For medical-related enquiries, please contact:

A/Prof Carolyn Ee; phone 0413 319 830; email: c.ee@westernsydney.edu.au

For all other enquiries, you can contact the study's coordinating principal investigator:

A/Prof Genevieve Steiner-Lim; phone 0410 342 397; email g.steiner@westernsydney.edu.au

Privacy Notice

Western Sydney University staff and students conduct research that may require the collection of personal and/or health information from research participants. The University's Privacy Policy and Privacy Management Plan set out how the University collects, holds, uses and discloses personal or health information. Further details about the use and disclosure of this information can be found on the Privacy at Western Sydney webpage.

What if I have a complaint?

If you have any complaints or reservations about the ethical conduct of this research, you may email the Ethics Committee through Research Services: humanethics@westernsydney.edu.au. Any issues you raise will be treated in confidence and investigated fully, and you will be informed of the outcome. If you agree to participate in this study, you may be asked to sign the Participant Consent Form. The information sheet is for you to keep, and the consent form is retained by the researcher/s.

This study has been approved by the Western Sydney University Human Research Ethics Committee. The Approval number is H[16200].

WESTERN SYDNEY
UNIVERSITY

Explanation of Consent

What will happen to my information if I agree to it being used in other projects?

Thank you for considering being a participant in a university research project. The researchers are asking that you agree to supply your information (data) for use in this project and to also agree to allow the data to potentially be used in future research projects.

This request is in line with current University and government policy that encourages the re-use of data once it has been collected. Collecting information for research can be an inconvenience or burden for participants and has significant costs associated with it. Sharing your data with other researchers gives potential for others to reflect on the data and its findings, to re-use it with new insight, and increase understanding in this research area.

You have been asked to agree to Extended consent.

What does this mean?

When you agree to extended consent, it means that you agree that your data, as part of a larger dataset (the information collected for this project) can be re-used in projects that are

- an extension of this project
- closely related to this project
- in the same general area of this research.

The researchers will allow this data to be used by members of the research team for similar projects. While the outcome of this research may inform future research projects, the actual data collected will not be utilized in future projects.

To enable this re-use, your data will be held at the University in its data repository and managed under a Data Management Plan. The stored data available for re-use will have information in it that makes you identifiable. The re-use of the data will only be allowed after an ethics committee has agreed that the new use of the data meets the requirements of ethics review.

The researchers want to keep the data for fifteen years for possible re-use. After this time the data will be securely destroyed.

You are welcome to discuss these issues further with the researchers before deciding if you agree. You can also find more information about the re-use of data in research in the National Statement on Ethical Conduct in Human Research – see Sections 2.2.14 - 2.2.18.

https://www.nhmrc.gov.au/about-us/publications/national-statement-ethical-conduct-human-research-2007-updated-2018

NICM Health Research Institute
Western Sydney University
Locked Bag 1797
Penrith NSW 2751
Australia

Ms Joelle Metri
Telephone: 0411 622 021
Email: j.metri@westernsydney.edu.au

Consent Form – Medical (Extended)

This consent form is not project specific. Participants baseline data may be used in future studies.

Project Title:

Exploring the Neurophysiological, Cognitive, Mood, and Biochemical Effects Associated with Non-Invasive Brain Stimulation During the Menopause Transition: A Randomised, Sham-Controlled, Double-Blinded, Pilot Controlled Clinical Trial (The MenoStim Trial)

This study has been approved by the Human Research Ethics Committee at Western Sydney University. The ethics reference number is: H[16200].

I hereby consent to participate in the above-named research project.

I acknowledge that:

☐ I have read the Participant Information Sheet (or where appropriate, have had it read to me) and have been given the opportunity to discuss the information and my involvement in the project with the researcher/s.

☐ The procedures required for the project and the time involved have been explained to me, and any questions I have about the project have been answered to my satisfaction.

☐ I consent to participating in this 5-week trial. I understand that remote pre-screening is expected to take up to half-an-hour including an online survey and a phone call.

☐ I understand that I will then be asked to visit a commercial DHM pathology practice to receive a blood test, which will require approximately half-an-hour of time.

☐ I understand that if I am enrolled in the trial, I will be expected to visit either the School of Health Sciences (Campbelltown) or the NICM Health Research Institute (Westmead) for 5 sessions of iTBS treatment over 1 week. I understand that each session will require approximately 1 hour.

☐ I understand that I will be asked to undergo testing at three timepoints (Day 1, Day 5, and Follow Up). Each testing session is expected to take approximately 3 hours.

☐ I understand that the total interactions with the research team over the 5-week period will be around 10–13 hours. I understand that I will be asked record any signs and symptoms I may feel in a diary throughout the course of the trial.

☐ I understand that I will be asked to complete surveys regarding my memory, thinking, and mood, and that a member of the research team will need to have close physical contact with me to complete some assessments.

☐ I understand that I will need to provide urine and saliva samples at three timepoints. I understand that my de-identified samples will be analysed. I understand that I will need to undergo electroencephalographic (EEG), transcranial magnetic stimulation (TMS), heart rate variability (HRV), skin conductance, and pressure-pain threshold testing at three timepoints.

☐ I understand that if I am eligible to be enrolled in this study that I will be randomly allocated to either the active treatment or the sham group. I understand that this allocation is unknown (blinded) to both me and the research team. I understand that after my involvement in this study that I will not have continued access to the study intervention.

☐ I understand that I will be asked to report any signs and symptoms I may feel over the course of the trial in an electronic diary managed by REDCap. I understand that REDCap is a secure web application that is used to build and manage online surveys and databases related to clinical trials. I understand that all adverse events that I report will be evaluated by the study physician.

☐ I will abide by COVID infection control measures as outlined by the researcher in accordance with current University policy and practices.

☐ I understand that my involvement is confidential, and that the information gained during the study may be published and stored for other research use but no information about me will be used in any way that reveals my identity but will only be used after additional ethical review.

☐ I understand that I can withdraw from the study at any time without affecting my relationship with the researcher/s, medical staff, the University, any organisations involved or my medical treatment, now or in the future.

☐ I consent for my data and information provided to be used in this project and other related projects for an extended period of time.

WESTERN SYDNEY UNIVERSITY

**Signed:**	
**Name:**	
**Date:**	

Return Address:

NICM Health Research Institute, Western Sydney University
Locked Bag 1797, Penrith NSW 2751, Australia

Ms Joelle Metri
Telephone: 0411 622 021
Email: j.metri@westernsydney.edu.au

Privacy Notice

Western Sydney University staff and students conduct research that may require the collection of personal and/or health information from research participants.

The University's Privacy Policy and Privacy Management Plan set out how the University collects, holds, uses and discloses personal or health information. Further details about the use and disclosure of this information can be found on the Privacy at Western Sydney webpage.

What if I have a complaint?

If you have any complaints or reservations about the ethical conduct of this research, you may email the Ethics Committee through Research Services: humanethics@westernsydney.edu.au.

Any issues you raise will be treated in confidence and investigated fully, and you will be informed of the outcome.
