# Supplementary material for: The MenoStim Trial: Study Protocol for a Randomised, Sham-Controlled, Double-Blinded, Pilot Clinical Trial Exploring the Neurophysiological, Cognitive, Mood and Biochemical Effects Associated with Non-Invasive Brain Stimulation During the Menopause Transition
Source: BMJ Open. 2025 Dec 19;15(12):e106745. doi: 10.1136/bmjopen-2025-106745 (PMC12716591; doi:10.1136/bmjopen-2025-106745)
Supplement: Supplementary file 4 [file bmjopen-2025-106745.reviewer_comments.pdf]

PEER REVIEW HISTORY

BMJ Open publishes all reviews undertaken for accepted manuscripts. Reviewers are asked to complete a checklist review form and are provided with free text boxes to elaborate on their assessment. These free text comments are reproduced below.

ARTICLE DETAILS

Title (Provisional)

Study Protocol for a Randomised, Sham-Controlled, Double-Blinded, Pilot Clinical Trial Exploring the Neurophysiological, Cognitive, Mood, and Biochemical Effects Associated with Non-Invasive Brain Stimulation During the Menopause Transition: The MenoStim Trial

Authors

Metri, Najwa-Joelle; Cavaleri, Rocco; Alhassani, Ghufran; Ee, Carolyn; Lim, Chai K.; Francis, Heather M.; Hochstrasser, Daniel; Bou Merhy, Rose Mery; Steiner-Lim, Genevieve Z.

VERSION 1 - REVIEW

**Reviewer**	**1**
**Name**	**Brunelin, Jérôme**
**Affiliation**	**CH Le Vinatier, PSYR2**
**Date**	**09-Oct-2025**
**COI**	**None**

This is a well-designed and timely protocol that has the potential to yield important insights in the case of positive outcomes. Nevertheless, the choice of sham stimulation with a 180-degree active coil may represent a methodological limitation. The sample size is well defined and seems adapted to the question. The study protocol and outcomes were pre registered in a public database/ The study was approved by an independant IRB/ This study was funded by a National Health and Medical Research Council with no role in publication and dissemination of results. The authors hva also provided the SPirit checklist as requested.

VERSION 1 - AUTHOR RESPONSE

Reviewer 1 Comments:

This is a well-designed and timely protocol that has the potential to yield important insights in the case of positive outcomes. Nevertheless, the choice of sham stimulation with a 180-degree active coil may

represent a methodological limitation. The sample size is well defined and seems adapted to the question. The study protocol and outcomes were pre-registered in a public database. The study was approved by an independent IRB. This study was funded by a National Health and Medical Research Council with no role in publication and dissemination of results. The authors have also provided the SPIRIT checklist as requested.

We thank the reviewer for their positive feedback and careful consideration of the study design. The sham stimulation method has been updated since the original submission. Rather than using a 180-degree coil rotation, we will now employ a dedicated sham coil that reproduces the audible clicks and superficial somatic sensations of active stimulation, without delivering magnetic pulses to the cortex. This approach is expected to provide a more credible sham condition and improve blinding integrity. Section 2.4 (Intervention; pp. 10, lines 210–213) have now been updated accordingly:

"Sham will be delivered using a dedicated sham coil that reproduces the audible clicks and superficial somatic sensations of active stimulation, without delivering magnetic pulses to the cortex. The set up will be handled by a blinded laboratory technician, and active and sham groups will receive identical information and instructions."
